# The Major Cow Milk Allergen Bos d 5 Manipulates T-Helper Cells Depending on Its Load with Siderophore-Bound Iron

**DOI:** 10.1371/journal.pone.0104803

**Published:** 2014-08-12

**Authors:** Franziska Roth-Walter, Luis F. Pacios, Cristina Gomez-Casado, Gerlinde Hofstetter, Georg A. Roth, Josef Singer, Araceli Diaz-Perales, Erika Jensen-Jarolim

**Affiliations:** 1 Comparative Medicine, Messerli Research Institute of the University of Veterinary Medicine Vienna, Medical University Vienna and University Vienna, Vienna, Austria; 2 Biotechnology Department, Center for Plant Biotechnology and Genomics, Technical University of Madrid, Madrid, Spain; 3 Department of Anesthesiology, General Intensive Care and Pain Medicine, Medical University of Vienna, Austria; 4 Comparative Immunology and Oncology, Department of Pathophysiology and Allergy Research, Center of Pathophysiology, Infectiology and Immunology, Medical University of Vienna, Vienna, Austria; Case Western Reserve University, United States of America

## Abstract

The mechanisms of allergic sensitization to milk are still elusive. The major allergen Bos d 5 belongs to the lipocalin-family and thus is able to transport numerous ligands. In this study we investigated its ability to bind to iron-siderophore complexes and tested the immune-modulatory properties of Bos d 5 in either forms. Structural and *in silico* docking analysis of Bos d 5 revealed that Bos d 5 is able to bind to iron via catechol-based flavonoids (quercetin, myricetin, luteolin) that act as siderophores as confirmed by spectral-analysis and iron staining. Calculated dissociation constants of docking analyses were below 1 µM by virtual addition of iron. When incubated with human peripheral blood mononuclear cells (PBMCs), only the *apo*-form of Bos d 5 led to an increase of CD4+positive cells and significantly elevated IL13 and IFNγ-levels. In contrast, *holo*-Bos d 5 decreased numbers of CD4 expressing cells and induced apoptosis. Taken together, our data give evidence that Bos d 5 is capable of binding iron via siderophores. Moreover, our data support for the first time the notion that the form of application (*apo*- or *holo*-form) is decisive for the subsequent immune response. The *apo*-form promotes Th2 cells and inflammation, whereas the *holo*-form appears to be immunosuppressive.

## Introduction

Cow milk allergy represents one of the most common food allergies affecting between 1.8 to 7.5% of infants during the first year of life [1]. In the majority of cases (85-90%) milk allergy resolve, however, children, who have outgrown milk allergy, still have a higher risk in developing atopic eczema, egg allergy or allergic asthma [2] Almost all known symptoms of food allergy (skin, gastrointestinal, respiratory) [3] including anaphylaxis have been reported after consumption of cow milk. Cow milk contains approximately 35 grams proteins per liter. About 80% of these proteins are caseins (80%) comprising αS1-, αS2-, β-, and κ-casein and the remaining 20% is present in the whey fractions consisting mainly of β-lactoglobulin (Bos d 5), α-lactalbumin and serum albumin. Caseins and β-lactoglobulin are regarded as the major allergens in milk. Because a Bos d 5 homologue is lacking in human milk, it has long been considered the most important cow's milk allergen.[4]

Bos d 5 belongs to the lipocalins, which are a functionally diverse family of proteins that in generally bind small, hydrophobic ligands. Despite their low sequence homology the core structure is highly conserved with an antiparallel-β barrel that defines the calyx [5]. Bos d 5, like most lipocalins, is thought to modulate cellular processes by binding to ligands. Numerous ligands have been reported to bind to Bos d 5 including fatty acids like palmitic acid with a dissociation constant, K_d_ of 0.6 µM or retinol having different affinities ranging from 0.6 µM [6] to 10 µM [7], [8]. Interestingly, Bos d 5 binds with similar or higher affinity to flavonols (K_d_ 0.3–1.5 µM) [9] with many possessing a catechol-core structure [6].

In this study, we aimed to investigate the immune-modulatory properties of Bos d 5. The fact that Bos d 5 is a major allergen implicates that at some point it is in contact with our immune system. Interestingly, also our human body harbors a great number of lipocalin proteins, some of which do have immune-regulatory function. In particular, the immune-modulatory protein human lipocalin-2, LCN2, draw our attention, by the facts that 1) it is highly expressed in sites exposed to the environment like the lung and the gut [10] and 2) that its immune-regulatory properties depend whether it carries iron via siderophores or not [11]-[13].

Siderophores are small, high-affinity iron chelating compounds and are amongst the strongest soluble iron(III) binding agents known, which usually are secreted by bacteria, fungi and grasses. The major groups of siderophores include the catecholates (phenolates), hydroxamates and carboxylates (e.g. derivates of citric acid) [14].

In this respect, we investigated the potential functions of the major allergen Bos d 5 to likewise bind to iron via siderophores and to act on primary human immune cells.

## Materials and Methods

### Materials

Bovine beta-lactoglobulin, deferoxamine mesylate, ammonium iron (III) citrate, potassium ferrocyanide, dihidroxybenzene (catechol), dihydroxybenzoic acid (DHBA) were all purchased from Sigma (Sigma Aldrich, Steinheim, Germany). Ficoll-Paque PLUS was obtained from GE Healthcare (Uppsala, Sweden). IFNγ and IL13 ELISA kits were from ebiosciences (Santa Clara, CA). Antibodies were either purchased by ebioscience (CD3-APC clone SK7; 7AAD) or BD Bioscience (CD4-PE-Cy7, clone SK3; CD8-PE, clone SK1; Annexin V FITC).

### Structural and Docking Analysis

Bos d 5 structure was taken from the complex with retinoic acid, PDB entry 1GX9 [15]. The geometries of ligand molecules were obtained upon energy minimization with the MM2 force field of structures drawn using the ChemBioDraw/ChemBio3D Ultra 12.0 package. Docking input files for protein and ligands were prepared with AutoDockTools [16]. A grid box of 22 Å x 22 Å x 22 Å with origin at a point corresponding to the C6 atom in the retinoic acid molecule in the 1GX9 structure was used for docking calculations which were performed with AutoDock Vina [17]. In all cases, the docking geometry with the lowest affinity energy *E_af_* was selected. Estimates of dissociation equilibrium constants *K_d_* were then calculated for the protein-ligand complexes by assuming Eaf ∼ ΔG with Kd  =  exp(-ΔG/RT) at *T*  =  298.15 K. Structural comparison of Bos d 5 with human NGAL (PDB entry 1L6M) was performed using FATCATflex (Flexible structure AlignmenT by Chaining Aligned Fragment Pairs) [18],

### Generation of *apo*-Bos d 5

Commercially available bovine beta-lactoglobulin was prior use three-times dialyzed against 10 µM deferoxamine mesylate solution. Subsequently, another dialyzation against deionized water was performed.

### Generation of *holo*-Bos d 5

*Apo*-Bos d 5 was incubated with an equimolar concentration of ammonium iron (III) citrate and a three-fold molar concentration of catechol.

### Binding assays

Optical spectra of siderophore-iron complex containing 375 µM iron and 1.125 mM were monitored in the presence or absence of 362 µM Bos d 5 in Tris-buffer at pH 7. Control spectra only contained Bos d 5. Absorbance was measured between 280 and 950 nm in 5 nm increments in quarz cuvettes using the spectrophotometer (TECAN infinite200Pro, Tecan, Crailsheim, Germany).

For iron staining 5 µl of the above-mentioned solutions (*apo*- and *holo-*Bos d 5, Fe-DHBA-complex as well as buffer control) were dotted on a nitrocellulose membrane. After rigorous washing in 20 mM Tris-buffer containing 0.5% Tween-20, membrane was stained with 5% potassium ferrocyanide in 1 M HCl for 20 minutes. Membrane was washed with water and iron-staining (Prussian blue) on dry membrane was visualized using incident light with a FluorChem FC3 apparatus (proteinsimple, Santa Clara, California).

### Isolation of PBMCs

The study was approved by the institutional ethics committee of the Medical University of Vienna and conducted in accordance with the Helsinki Declaration of 1975. Twenty-five volunteers donated 15 ml blood. All subjects gave their full written informed consent.

Blood was mixed with equal volumes of PBS containing 2% fetal calf serum (FCS) before applying onto 10 ml Ficoll-Paque PLUS, centrifugation at 400 g for 30 min without brake and washing the cells twice with 0,89% sodium chloride solution. Cells were then diluted to a concentration of 1 Mio cells/ml in RPMI medium containing 10% FCS. Throughout the study FCS of the same lot was used.

### Stimulation of PBMCs

Isolated PBMCs (0.5 Mio/test) were incubated with a final concentration of 0.75 ng/ml PMA, 100 µg/ml *apo*-Bos d 5 in the presence or absence of 30 µM catechol and 10 µM iron. Controls included PMA alone or in the presence of 30 µM catechol and 10 µM iron. PMA concentration was determined in pre-experiments and considered optimal when cells were slightly downregulating surface CD4+ expression [19]. After 18 h supernatants were collected and stored at -80°C until further analysis.

Cells were stained for 30 min at 4°C with CD3-APC, CD4-PE-Cy7, CD8-PE in PBS containing 2% FCS, followed by 10 min incubation of Annexin V FITC and 7AAD in Binding buffer (10 mM Hepes, 140 mM NaCl, 2.5 mM CaCl_2_) at room temperature. Acquisition and analysis was performed on a FACS Canto II machine (BD Bioscience, San Jose, CA, USA) using the FACSDiva Software 6.0. Living Cells were gated by forward and sideward scatter. Further gating was performed on CD3 cells, before plotting towards CD4 and CD8 were performed. For Annexin V staining, living cells were plotted for CD4 and Annexin V without further gating to CD3.

### Determination of Cytokines

IL13 and IFNγ were detected with commercially available kits according to the manufacturers' protocol using undiluted supernatants. ELISAs for human IL13 and IFNγ have a reported sensitivity of 4 pg/ml.

### Statistical Analysis

Statistical analyses were conducted with repeated measures ANOVA following Newman-Keuls Multiple Comparison test was calculated using GraphPad Prism 5 software (GraphPad, San Diego, CA, USA). *P*<0.05 was considered statistically significant.

## Results

### Bos d 5 is able to bind to iron-siderophore complexes

Structural alignment using the FATCATflex approach revealed a high structural homology of Bos d 5 (PDB entry 1GX9) with human LCN2 (PDB entry 1L6M) as demonstrated by FATCATflex superposition parameters (p = 7.64×10^-12^, Score 310.66. RMSD: 3.14, data not shown).

For docking experiments same crystal structure of Bos d 5 was taken for protein while the following compounds were selected for the ligands: DHBA chosen as a simple model of catechol-based siderophores and flavonoids quercetin, luteolin and myricetin known to be present in fruits, vegetables, leaves and grains [20], [21]. Retinol and retinoic acid were also taken as control ligands (Table 1). Docking analyses were also performed with virtual addition of Fe to DHBA and flavonoids [22]. Indeed, we were able to support the notion that flavonoids are siderophores and that affinities of Bos d 5 actually improved by virtual addition of Fe. Dissociation constants estimated for quercetin from docking results changed from 1.4 µM in the absence of Fe to 0.51 and 0.11 µM for one and two quercetin molecules ligating iron, respectively. For luteolin these variations were 1.0 to 0.61 to 0.078 µM, myricetin 1.0 to 0.72 to 0.22 µM. DHBA affinities also improved from 43 µM for isolated DHBA to 31 for Fe(DHBA) to 0.85 µM for Fe(DHBA)_2_. In comparison the known ligands of Bos d 5 retinol and retinoic acid displayed dissociation constants of 0.51 and 1.7 µM, respectively. Hence, our results suggest that Bos d 5 is able to transport iron via flavonoids with affinities exceeding the binding of retinol and retinoic acid (Table 1).

**Table 1:** Calculated affinities energies and dissociation constants of ligands to Bos d 5.

Ligand	Affinity energy (kcal/mol)	Dissociation constant K_d_ (µM)
Quercetin	−7.9	1.4
Fe-(Quercetin)	−8.5	0.51
Fe-(Quercetin)_2_	−9.4	0.11
Luteolin	−8.1	1.0
Fe-(Luteolin)	−8.4	0.61
Fe-(Luteolin)_2_	−9.6	0.078
Myricetin	−8.1	1.0
Fe-(Myricetin)	−8.3	0.72
Fe-(Myricetin)_2_	−9.0	0.22
DHBA	−5.9	43
Fe-(DHBA)	−6.1	31
Fe-(DHBA)_2_	−8.2	0.85
Fe-(DHBA)_3_	−7.0	6.6
retinol	−8.5	0.51
retinoic acid	−7.8	1.7

**Figure 1:**
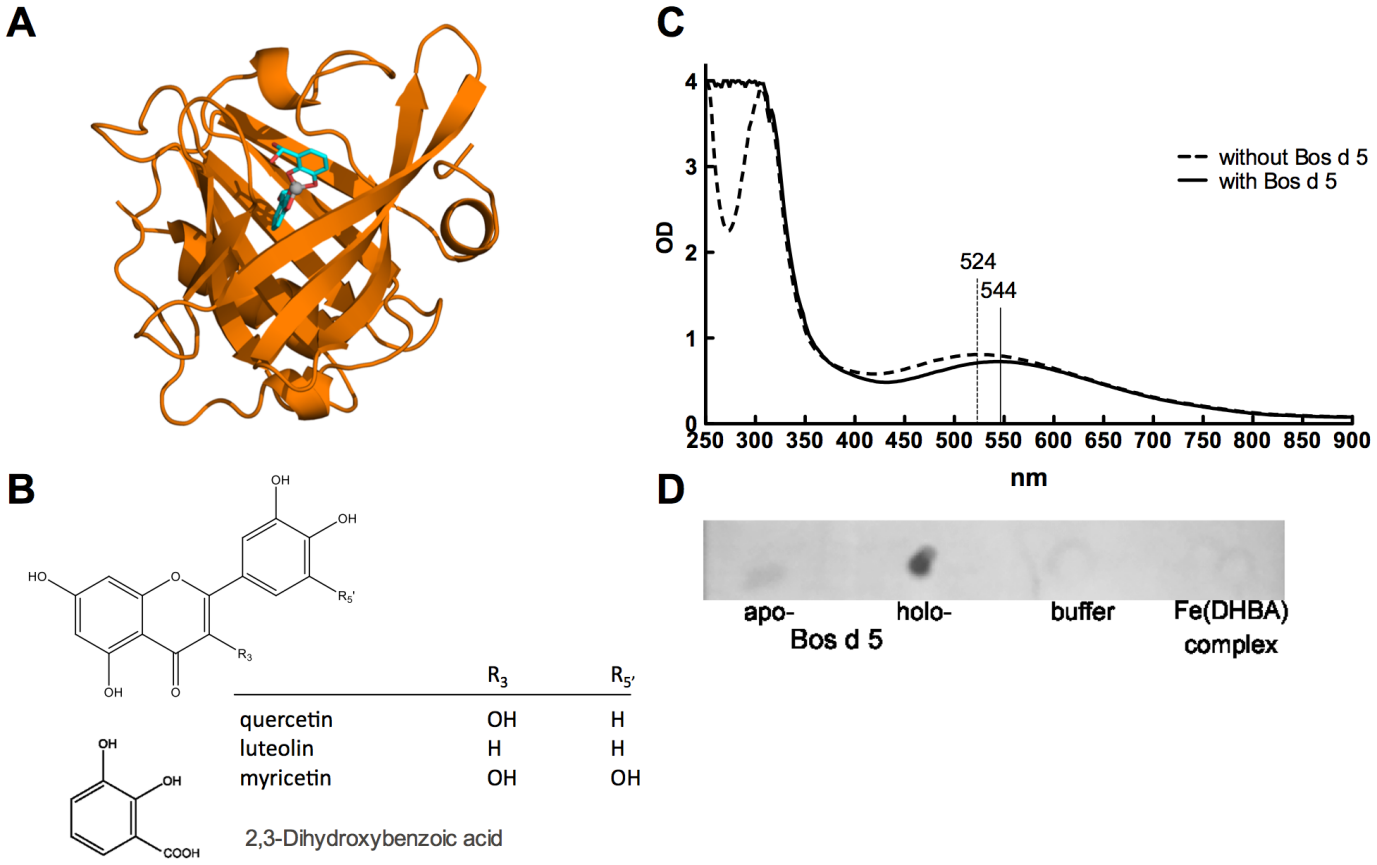
Bos d 5 is able to bind iron via siderophores. (A) Bos d 5 (1GX9) with Fe(DHBA)_2_ (B) chemical structure of siderophores (C) UV-VIS spectra of Fe(DHBA) complex in the presence or absence of Bos d 5 or with Bos d 5 alone. (D) Iron-staining of *apo*- or *holo*-Bos d 5 as well as controls (buffer and iron-siderophore-complex) dotted on nitrocellulose-membrane.

Iron-binding capacity of Bos d 5 was also monitored by acquisition of UV-visible spectra of the siderophore-iron complex alone or in the presence of Bos d 5. Binding of the siderophore-iron-complex to Bos d 5 resulted in a shift in the visible spectra from 524 to 544 nm (Fig. 1C). Moreover, iron staining with Prussian blue revealed that indeed iron was found bound to Bos d 5 (Fig. 1D). For excluding unspecific binding of the siderophore-iron complex alone to the membrane, complex and buffer controls were included. Hence, our *in vitro* and *in silico* data suggest that Bos d 5 has the potential to bind iron via catechol-based siderophores.

### *Apo*- but not *holo*- Bos d 5 promote Th2 cells

**Figure 2:**
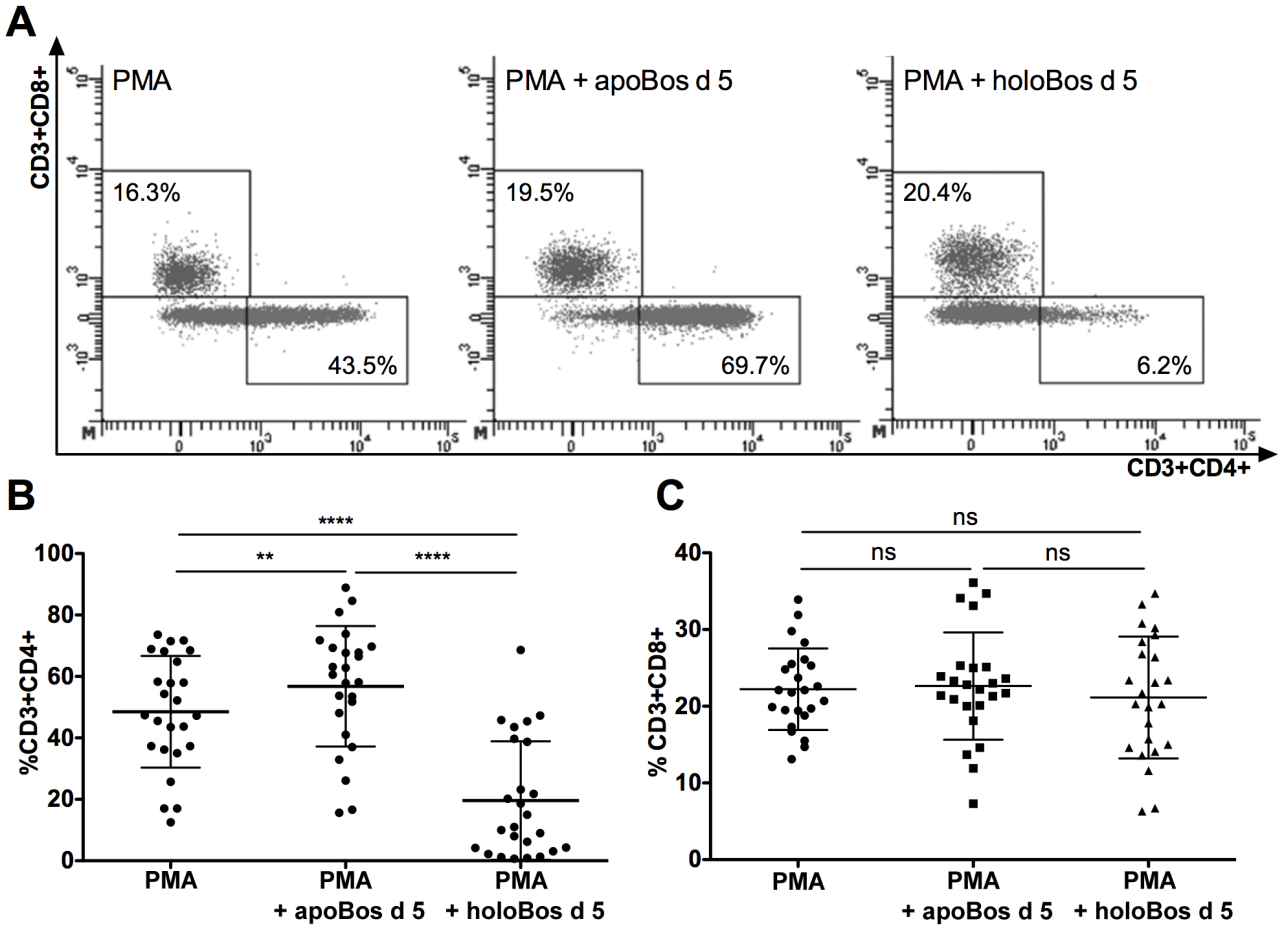
*Apo*-, but not *holo*-Bos d 5 promotes CD4 cells. PBMCs were treated with PMA alone or in the presence of *apo*- or *holo-*Bos d 5. (A) Living cells were gated on forward and side scatter, before gating on CD3 positive cells were performed. CD3 gated cells were then analyzed for their CD4 and CD8 expression.Representative pictograms of CD3 gated PBMCs plotted for CD4 and CD8 (B) Percentage of CD3+CD4+ cells. (C) Percentage of CD3+CD8+ cells. Statistical analyses were conducted with repeated measures ANOVA following Newman-Keuls Multiple Comparison test. **P<0.01, ****P<0.0001, ns not significant.

**Figure 3:**
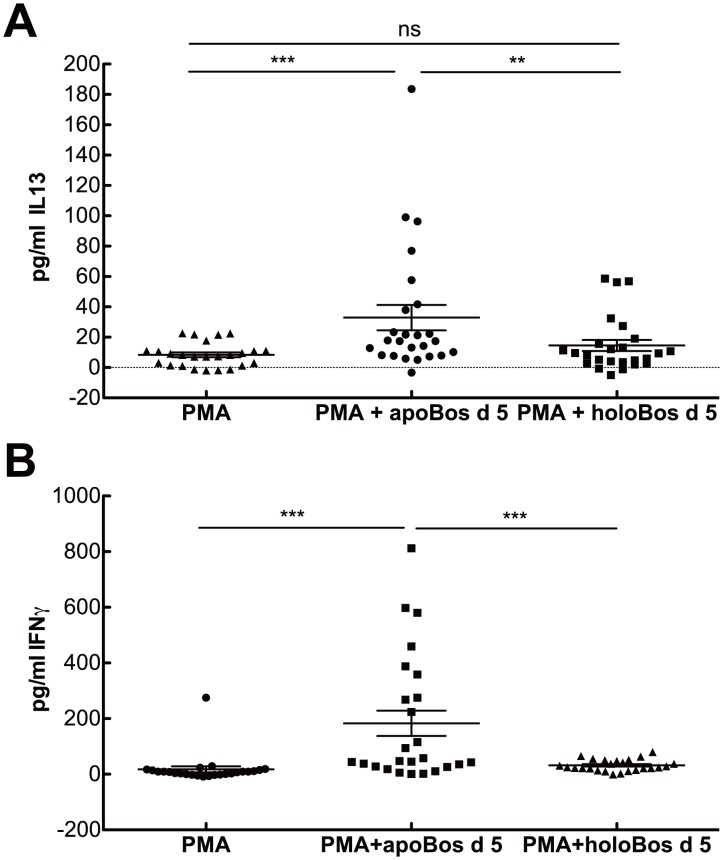
Bos d 5 devoid of iron promotes IL13 and IFNγ secretion. (A) IL13 levels and (B**)** IFNγ levels of stimulated PBMCs. Statistical analyses were conducted with repeated measures ANOVA following Newman-Keuls Multiple Comparison test. **P<0.01, *** P<0.001, ns not significant.

LCN2 is able to drive immune responses dependent on its iron-loading state [11]. Consequently, in our next approach we investigated whether primary human immune cells discerned between the *apo*- (without iron-siderophore) and the *holo*-state (with iron-siderophore) of Bos d 5. Indeed, activation of peripheral blood mononuclear cells, PBMCs, from healthy individuals with low concentration of phorbol12-myristate13-acetate, PMA, and *apo*- or *holo*-Bos d 5 for 18 h *in vitro* resulted in a completely different CD4 expression pattern of CD3+ cells as well as cytokine profiles. Stimulation of immune cells with *apo*-Bos d 5 promoted expression of CD3+CD4+ lymphocytes, whereas introduction of the *holo*-form further decreased CD4+ expression in lymphocytes (Fig. 2A,B). Interestingly, Bos d 5 only had an impact on CD4, but not on the CD8 expression (Fig. 2C). To further determine which type of Thelper cells were promoted by stimulation of human immune cells with the *apo*-form of Bos d 5, cytokine contents in supernatants were analyzed for typical Th1 (IFNγ) and Th2 (IL13) cytokines. Indeed, we were able to detect IL13 already after 18 h stimulation with *apo*-Bos d 5 in PMA-activated PBMCs suggesting that Th2-cells were generated (Fig.3), which was not seen when cells were incubated with the *holo*-form. However, also IFNγ were generated upon addition of *apo*-Bos d 5, but not upon addition of the *holo*-form, suggesting that Th1-cells were the source of IFNγ (Fig. 3).

**Figure 4:**
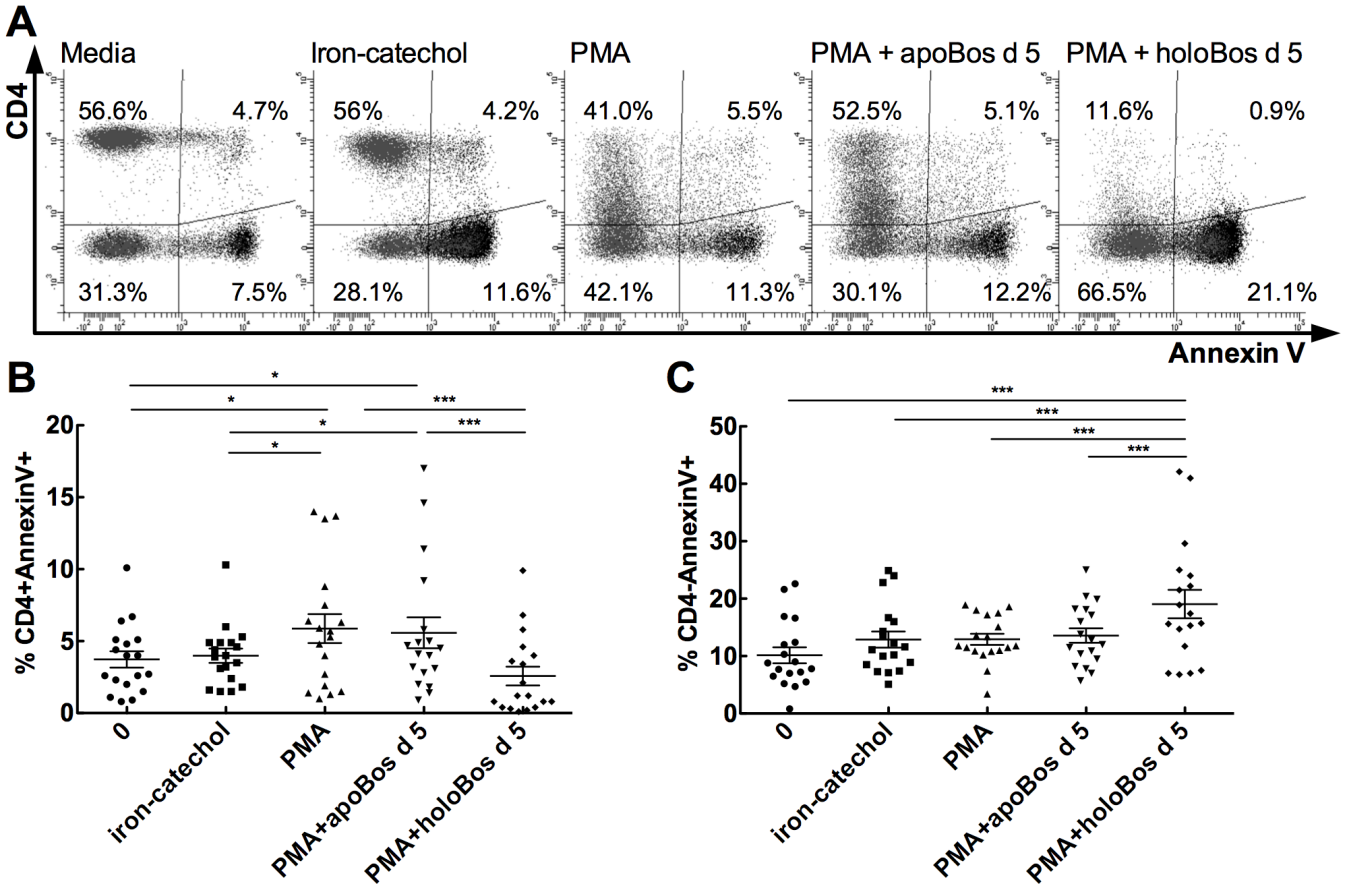
Annexin V staining of PBMCs. PBMCs were left unstimulated or treated with iron-catechol alone or with PMA alone or in the presence of *apo*- or *holo*-Bos d 5. Living cells were gated on forward and side scatter and then plotted for CD4 and Annexin V. (A) Representative pictograms of PBMCs plotted for CD4 and Annexin V. (B) Summary of percentage of CD4+Annexin V+ cells. (C) Summary of percentage of CD4-Annexin V+ cells. Statistical analyses were conducted with repeated measures ANOVA following Newman-Keuls Multiple Comparison test. ***P<0.001, *P<0.05.

Cells were also stained for Annexin V. Careful analysis revealed that no significant changes in Annexin V staining could be observed in untreated (media) or with iron-catechol incubated controls. In contrast significant more CD4+, but not CD4-negative cells could be stained for Annexin V upon incubation with PMA (Fig. 4). Further addition of *apo*-Bos d 5 did not alter Annexin V-staining of PMA-stimulated cells. In contrast addition of *holo*-Bos d 5 to PMA-stimulated PBMCs rendered significantly less Annexin V+ and CD4+ positive cells than Annexin V+ CD4-negative cells (Fig. 4). Hence, cells appeared to downregulate CD4-expression before undergoing apoptosis upon incubation with the *holo*-version of Bos d 5 (Fig.4). Thus, Bos d 5 was able to mount a Th2-response in its *apo*-form, but it abrogated an immune response partly by induction of apoptosis when introduced to immune cells in its *holo*-form.

## Discussion

Milk allergy is one of the most common food allergies. Most children will outgrow their allergy within the first years, however they pertain a greater risk to develop respiratory allergies like hay fever or asthma. Indeed, between 50-80% of milk allergic infants will develop respiratory allergies before puberty [23].

This is interesting, because most respiratory allergens deriving from mammalians, and recently also the major birch pollen allergen Bet v 1 [24], were identified to belong to the same protein family as the major milk allergen Bos d 5, namely the lipocalins. Lipocalins share a common tertiary structure consisting of a β-barrel enclosing an internal ligand binding sites, but have very low sequence homology [25]. They are extracellular proteins whose expression is confirmed in bacteria, protoctists, plants, arthropods and chordates [26]. The humans homologue is termed LCN2. In respect to xenogenous lipocalins in general, one striking feature that they do seem to have in common is their very low immunogenicity, despite their high allergenic potential [27].

Thus, we hypothesized that normally Bos d 5 harbors immune-suppressive qualities, but under certain conditions could be able to interfere with immune-regulatory proteins like human LCN2, thereby skewing the system towards Th2.

The biological function of Bos d 5 in milk is still not clear. It is known to bind to retinol and retinoic acid in the µM range, but interestingly docking analysis revealed similar or even stronger binding to flavonols from plants (K_d_ = 0.2–0.6 µM) with many possessing a catechol-core structure. The possible transfer of flavonols to milk was confirmed in a recent study showing that phenolic compounds from plants were found in the milk of grazing cows [28]. An important aspect hereby is that flavonoids are known metal-chelators and thus have stronger free radical scavenging properties than free flavonoids [29]. Support of Bos d 5 carrying iron also comes from research using β-lactoglobulin hydrolysate-iron complexes as a vehicle for iron supplementation [30] and showing that iron-binding properties of Bos d 5 *per se* exceeded that of α-lactalbumin [31].

Thus, binding of Bos d 5 to plant-derived flavonoids and polyphenols complexed with iron is a possible scenario happening under steady-state situations in the milk.

Our *in silico* as well as *in vitro* data moreover support the notion that Bos d 5 is able to bind to siderophores in conjunction with iron in affinities similarly seen with retinol. It is unlikely that retinol itself is able to bind iron due to the lack of neighboring hydroxyl-groups or of a hydroxyl-group adjacent to a carbonyl-group, even though a thorough search in the literature revealed that retinol, which itself is important during immune-homeostasis and inflammation, is linked with iron-metabolisms [32]–[34]. Moreover, it is striking to note that many lipocalin-proteins bind to retinol [35] and only in recent years their ability to bind siderophores has been discovered. We were however methodologically unable to address whether a direct connection between retinol and ferric ions exists.

Analogously, there are multiple studies showing that the human counterpart LCN2 binds to catechol-based structures, when they are complexed with iron [22], [36]. Moreover, for LCN2 the biological function clearly is dictated whether it binds iron or not [11], [12], [37], [38].

In this respect, we deprived commercially available Bos d 5 of iron (*apo*-form) and on purpose loaded part of it with iron, before adding it in their *apo*- or *holo*-form to activated isolated immune cells of human subjects.

Indeed, we show here for the first time that Bos d 5 acts distinctly on immune cells dependent whether it is introduced in its *apo*- or *holo*-form. *Apo*-Bos d 5 per se was able to mount a Th2 response leading to increased numbers of CD4+ cells and the secretion of IL13 as well as IFNγ. Since we analyzed serum of a mixed immune cells population, we cannot exclude that the cytokines derived from other immune cell population, e.g. IFNγ from NK cells, Th1 and cytotoxic T cells and IL13 from Th2 cells, but also group 2 innate lymphoid cells including natural helper cells and nuocytes [39]. The parallel induction of a Th1 as well as a Th2 response is a common phenomenon [27], [40], [41]. Moreover, in allergics the ratio between Th1/Th2 cells seems rather to reflect the allergic status than the presence of Th1 cells alone [41]. Importantly, in our study the *holo*-version of Bos d 5 had an antagonistic effect to the *apo-*form and resulted in a reduction of CD4+ cell numbers and their increased cell death, without any significant cytokine secretion.

The obtained results have significant implications in understanding the pathophysiological mechanisms leading to allergic sensitization. These results suggest that Bos d 5 - under natural conditions - is found in its *holo*-form and as such generally suppresses an immune response. We speculate that in situations in which the local mucosal environment has an increased need for iron, e.g. during bacterial infections by bacterial siderophores or during inflammation, Bos d 5 can display all its allergenic properties in turning into its *apo*-form.

Indirect evidence is given in a study linking maternal illnesses during pregnancy with an increased risk of asthma at 5 years [42]. Further, cow feed, organic farming, or milk processing and formula production may have significant impact on the allergenicity of Bos d 5 by influencing its iron load. For instance, milk from cows fed fresh green forage will have a higher proportion of unsaturated fatty acids [43] as well increased phenolic compounds [28] compared to cows on silage diet. Moreover, homogenization not only reduces the milk fat globules size to less than 1.0 µm, but has been shown to lead to denaturation of whey proteins in particular in pasteurized and in a lesser extend also in raw milk [44]. Similary, pasteurization has been shown to cause aggregtion of whey proteins [27], as well as to decrease copper and iron content [45] in milk.

In this respect, it is also noteworthy to mention that farm milk consumption is inversely associated with asthma and allergy and was independent of farm-related co-exposures in a cross sectional multi-center study including 14893 children aged 5–13 years [46]. Hence, the overall composition as well as food processing procedure will influence the allergenic potential of milk allergens.

In conclusion, we provide evidence that Bos d 5 is capable of binding iron via siderophores with similar or higher affinities as retinol. Moreover, our data attest that the iron load of Bos d 5 is decisive for the subsequent immune response with the *apo*-form skewing the system towards Th2, whereas the *holo*-form being immunosuppressive.
